# Supplementary material for: Computer aided detection for laterally spreading tumors and sessile serrated adenomas during colonoscopy
Source: PLoS One. 2020 Apr 21;15(4):e0231880. doi: 10.1371/journal.pone.0231880 (PMC7173785; doi:10.1371/journal.pone.0231880)
Supplement: Attachment — Submitted filename: response to reviewers.docx [file pone.0231880.s005.docx]

Please include the following items when submitting your revised manuscript:

- A rebuttal letter that responds to each point raised by the academic editor and reviewer(s). This letter should be uploaded as separate file and labeled 'Response to Reviewers'.
- A marked-up copy of your manuscript that highlights changes made to the original version. This file should be uploaded as separate file and labeled 'Revised Manuscript with Track Changes'.
- An unmarked version of your revised paper without tracked changes. This file should be uploaded as separate file and labeled 'Manuscript'.

When submitting your revision, we need you to address these additional requirements.

1. Please ensure that your manuscript meets PLOS ONE's style requirements, including those for file naming. The PLOS ONE style templates can be found at
   [http://www.journals.plos.org/plosone/s/file?id=wjVg/PLOSOne_formatting_sample_main_body.pdf](http://www.journals.plos.org/plosone/s/file?id=wjVg/PLOSOne_formatting_sample_main_body.pdf) and [http://www.journals.plos.org/plosone/s/file?id=ba62/PLOSOne_formatting_sample_title_authors_affiliations.pdf](http://www.journals.plos.org/plosone/s/file?id=ba62/PLOSOne_formatting_sample_title_authors_affiliations.pdf)

Answer: we have modified the manuscript according to PLOS ONE style.

2. We suggest you thoroughly copyedit your manuscript for language usage, spelling, and grammar. If you do not know anyone who can help you do this, you may wish to consider employing a professional scientific editing service.

Whilst you may use any professional scientific editing service of your choice, PLOS has partnered with both American Journal Experts (AJE) and Editage to provide discounted services to PLOS authors. Both organizations have experience helping authors meet PLOS guidelines and can provide language editing, translation, manuscript formatting, and figure formatting to ensure your manuscript meets our submission guidelines. To take advantage of our partnership with AJE, visit the AJE website ([http://learn.aje.com/plos/](http://learn.aje.com/plos/)) for a 15% discount off AJE services. To take advantage of our partnership with Editage, visit the Editage website ([www.editage.com](http://www.editage.com)) and enter referral code PLOSEDIT for a 15% discount off Editage services.  If the PLOS editorial team finds any language issues in text that either AJE or Editage has edited, the service provider will re-edit the text for free.

Upon resubmission, please provide the following:
•    The name of the colleague or the details of the professional service that edited your manuscript
•    A copy of your manuscript showing your changes by either highlighting them or using track changes (uploaded as a *supporting information* file)
•    A clean copy of the edited manuscript (uploaded as the new *manuscript* file)

Answer: Thanks for the recommendation. We’ve made some edits to the manuscript, hopefully it is now with decent English. If language is the last problem there, we can have it further finetuned.

2. In the ethics statement in the Methods and online submission information, please ensure that you have specified the form of consent that you obtained (for instance, written or verbal, and if verbal, how it was documented and witnessed).

Written informed consents were obtained from all participants, as modified in the revised manuscript

3. Thank you for stating the following in the Competing Interests section: "I have read the journal's policy and the authors of this manuscript have the following competing interests:The CADe system (EndoScreener) was developed by Shanghai Wision AI Co., Ltd. The system was provided free-of-charge for this study. Employees in the company were not involved in the study in any way, including in study design, statistical analysis or manuscript writing."

We have added this section according to your requirement

Please confirm that this does not alter your adherence to all PLOS ONE policies on sharing data and materials, by including the following statement: "This does not alter our adherence to  PLOS ONE policies on sharing data and materials.” (as detailed online in our guide for authors [http://journals.plos.org/plosone/s/competing-interests](http://journals.plos.org/plosone/s/competing-interests)).  If there are restrictions on sharing of data and/or materials, please state these. Please note that we cannot proceed with consideration of your article until this information has been declared.

Please include your updated Competing Interests statement in your cover letter; we will change the online submission form on your behalf.

We have added the statements in the manuscript according to your requirement

Please know it is PLOS ONE policy for corresponding authors to declare, on behalf of all authors, all potential competing interests for the purposes of transparency. PLOS defines a competing interest as anything that interferes with, or could reasonably be perceived as interfering with, the full and objective presentation, peer review, editorial decision-making, or publication of research or non-research articles submitted to one of the journals. Competing interests can be financial or non-financial, professional, or personal. Competing interests can arise in relationship to an organization or another person. Please follow this link to our website for more details on competing interests: [http://journals.plos.org/plosone/s/competing-interests](http://journals.plos.org/plosone/s/competing-interests)

5. In your Data Availability statement, you have not specified where the minimal data set underlying the results described in your manuscript can be found. PLOS defines a study's minimal data set as the underlying data used to reach the conclusions drawn in the manuscript and any additional data required to replicate the reported study findings in their entirety. All PLOS journals require that the minimal data set be made fully available. For more information about our data policy, please see [http://journals.plos.org/plosone/s/data-availability.](http://journals.plos.org/plosone/s/data-availability.)

Upon re-submitting your revised manuscript, please upload your study’s minimal underlying data set as either Supporting Information files or to a stable, public repository and include the relevant URLs, DOIs, or accession numbers within your revised cover letter. For a list of acceptable repositories, please see [http://journals.plos.org/plosone/s/data-availability#loc-recommended-repositories.](http://journals.plos.org/plosone/s/data-availability#loc-recommended-repositories.) Any potentially identifying patient information must be fully anonymized.

Important: If there are ethical or legal restrictions to sharing your data publicly, please explain these restrictions in detail. Please see our guidelines for more information on what we consider unacceptable restrictions to publicly sharing data: [http://journals.plos.org/plosone/s/data-availability#loc-unacceptable-data-access-restrictions.](http://journals.plos.org/plosone/s/data-availability#loc-unacceptable-data-access-restrictions.) Note that it is not acceptable for the authors to be the sole named individuals responsible for ensuring data access.

We will update your Data Availability statement to reflect the information you provide in your cover letter.

We have added a data availability section to declare where the minimal and entire data set underlying the results described in the manuscript can be found

6. Please amend either the title on the online submission form (via Edit Submission) or the title in the manuscript so that they are identical.

We have confirmed the title as “Computer aided detection for laterally spreading tumors and sessile serrated adenomas during colonoscopy”

Reviewers' comments:

Reviewer's Responses to Questions

**Comments to the Author**

1. Is the manuscript technically sound, and do the data support the conclusions?

The manuscript must describe a technically sound piece of scientific research with data that supports the conclusions. Experiments must have been conducted rigorously, with appropriate controls, replication, and sample sizes. The conclusions must be drawn appropriately based on the data presented.

Reviewer #1: Yes

2. Has the statistical analysis been performed appropriately and rigorously?

Reviewer #1: I Don't Know

 Answer: the statistical analysis of the study is a basic validation of sensitivity and specificity, these are very routine metrics, the definitions are consistent with our previous study which published at “*Nature Biomedical Engineering 2018;2:741–748*”, the methodology and definitions are also consistent with other major studies around AI-medicine filed. etc.

*Wang P, Xiao X, Glissen Brown JR, et al. Development and validation of a deep-learning algorithm for the detection of polyps during colonoscopy. Nature Biomedical Engineering 2018;2:741–748*

*Bernal, J. et al. Comparative validation of polyp detection methods in video colonoscopy: results from the MICCAI 2015 endoscopic vision challenge. IEEE Trans. Med. Imaging 36, 1231–1249 (2017).*

*Bandos AI, Rockette HE, Song T, Gur D. Area under the free response ROC curve (FROC) and a related summary index. Biometrics 2009;65:247–256*

3. Have the authors made all data underlying the findings in their manuscript fully available?

The [PLOS Data policy](http://www.plosone.org/static/policies.action#sharing) requires authors to make all data underlying the findings described in their manuscript fully available without restriction, with rare exception (please refer to the Data Availability Statement in the manuscript PDF file). The data should be provided as part of the manuscript or its supporting information, or deposited to a public repository. For example, in addition to summary statistics, the data points behind means, medians and variance measures should be available. If there are restrictions on publicly sharing data—e.g. participant privacy or use of data from a third party—those must be specified.

Reviewer #1: Yes

4. Is the manuscript presented in an intelligible fashion and written in standard English?

PLOS ONE does not copyedit accepted manuscripts, so the language in submitted articles must be clear, correct, and unambiguous. Any typographical or grammatical errors should be corrected at revision, so please note any specific errors here.

Reviewer #1: Yes

5. Review Comments to the Author

Please use the space provided to explain your answers to the questions above. You may also include additional comments for the author, including concerns about dual publication, research ethics, or publication ethics. (Please upload your review as an attachment if it exceeds 20,000 characters)

Reviewer #1: Colonoscopy is the gold standard for detecting and removing lesions to prevent cancer. Unfortunately, the miss rate can be as high as 25% even for adenomatous polyps and even higher for LST and SSA/P. The authors have developed an automated system to detect lesions. They test whether this system permits LST and SSA/P to be reliably detected. The data indicate the system improves detection but still needs to be perfected. A few concerns need to be addressed.

Major

1) For a broad audience, much more information needs to be provided regarding the basics of colorectal cancer and image analysis. For example, a figure showing different pathologies would be invaluable.

Answer: thanks for the comments. For a broad audience, we added a background introduction at the beginning of the discussion section, as “CRC is one of the leading causes of cancer related death[^[1]^] [^[2]^] and is a major public health issue given its high incidence and mortality rate. Screening colonoscopies have allowed a significant decrease of mortality rate and incidence of CRC in adults (by 51% and 32%, respectively). colonoscopy has also allowed an increase in 5-year survival rate in CRC, mainly as a result of early detection as well as removal of precancerous lesion”

and a figure 3 which illustrates the detection performance of CADe system on LSTs with different pathologies.

Fig 3

2) Define all terms and abbreviations when first used.
Answer: we confirm all terms and abbreviations were defined

3) The Discussion should address how this work could impact clinical practice.
Answer: we added “Noteworthy, although partially tagged, the alerts generated by the CADe system may still effectively notice the endoscopist and avoid a miss during real clinical setting. ” to show the impact of the CADe system on LSTs detection during colonoscopy; we added “Nevertheless, the performance was consistent with the previous technical study, the same CADe system with such a sensitivity for SSA/Ps has increased the deetction rate of diminutive polyps significantly” to show the impact of the CADe system on SSA/Ps detection during colonoscopy

Minor

1) Intro - "alone" should be "along"
2) Intro - "literatures" should be "studies"
3) Methods - averagely is not a word
4) Discussion - "caner" should be "cancer"

All the typos have been corrected

6. PLOS authors have the option to publish the peer review history of their article ([what does this mean?](https://journals.plos.org/plosone/s/editorial-and-peer-review-process#loc-peer-review-history)). If published, this will include your full peer review and any attached files.


If you choose “no”, your identity will remain anonymous but your review may still be made public.


**Do you want your identity to be public for this peer review?** For information about this choice, including consent withdrawal, please see our [Privacy Policy](https://www.plos.org/privacy-policy).

Reviewer #1: No


[NOTE: If reviewer comments were submitted as an attachment file, they will be attached to this email and accessible via the submission site. Please log into your account, locate the manuscript record, and check for the action link "View Attachments". If this link does not appear, there are no attachment files to be viewed.]


While revising your submission, please upload your figure files to the Preflight Analysis and Conversion Engine (PACE) digital diagnostic tool, [https://pacev2.apexcovantage.com/](https://pacev2.apexcovantage.com/). PACE helps ensure that figures meet PLOS requirements. To use PACE, you must first register as a user. Registration is free. Then, login and navigate to the UPLOAD tab, where you will find detailed instructions on how to use the tool. If you encounter any issues or have any questions when using PACE, please email us at [figures@plos.org](mailto:figures@plos.org). Please note that Supporting Information files do not need this step.

1. ^[]^ American Cancer Society. Cancer Facts and Figures: 2017. Atlanta, Georgia. 2017. <https://www.cancer.org/research/cancer-facts-statistics/all-cancer-facts-figures/>cancer-facts-fgures-2017.html. Accessed August 15, 2017
2. ^[]^ [Fang JY](https://www.ncbi.nlm.nih.gov/pubmed/?term=Fang%20JY%5BAuthor%5D&cauthor=true&cauthor_uid=26672726), [Zheng S](https://www.ncbi.nlm.nih.gov/pubmed/?term=Zheng%20S%5BAuthor%5D&cauthor=true&cauthor_uid=26672726), [Jiang B](https://www.ncbi.nlm.nih.gov/pubmed/?term=Jiang%20B%5BAuthor%5D&cauthor=true&cauthor_uid=26672726), et al. Consensus on the Prevention, Screening, Early Diagnosis and Treatment of Colorectal Tumors in China: Chinese Society of Gastroenterology, October 14-15, 2011, Shanghai, China. [Gastrointest Tumors](https://www.ncbi.nlm.nih.gov/pubmed/26672726) 2014;1:53-75
